# Long-term results of Gamma Knife Radiosurgery for Postsurgical residual or recurrent nonfunctioning Pituitary Adenomas

**DOI:** 10.7150/ijms.47168

**Published:** 2020-06-18

**Authors:** Yinhui Deng, Yanli Li, Xi Li, Lisha Wu, Tingting Quan, Chao Peng, Junyi Fu, Xin Yang, Jinxiu Yu

**Affiliations:** 1 Department of Radiotherapy, The Second Affiliated Hospital of Guangzhou Medical University, Guangzhou, Guangdong, China. 510260.; 2 Department of Endocrinology, The Second Affiliated Hospital of Guangzhou Medical University, Guangzhou, Guangdong, China. 510260.; 3 Department of Radiology, The Second Affiliated Hospital of Guangzhou Medical University, Guangzhou, Guangdong, China. 510260.; 4 Department of Medical Oncology, Sun Yat-sen Memorial Hospital, Sun Yat-sen University, Guangzhou, China. 510120.; 5 Department of Radiology, Sun Yat-sen University Cancer Center, State Key Laboratory of Oncology in South China, Collaborative Innovation Center for Cancer Medicine, Guangzhou, Guangdong, China, 510060.; 6 Department of Neurosurgery, Guangdong Provincial People's Hospital, Guangdong Academy of Medical Sciences, Guangzhou, Guangdong, China. 510080.; 7 Department of Neurology, The Second Affiliated Hospital of Guangzhou Medical University, Guangzhou, Guangdong, China. 510260.; 8 Department of Thoracic Surgery, The Second Affiliated Hospital of Guangzhou Medical University, Guangzhou, Guangdong, China. 510260.

**Keywords:** Gamma Knife, radiosurgery, nonfunctioning, pituitary adenoma

## Abstract

**Introduction:** The aim of this retrospective study was to analyze the long-term outcomes and factors associated with treatment failure of Gamma Knife radiosurgery (GKRS) for postsurgical residual or recurrent nonfunctioning pituitary adenomas (NFPAs).

**Design and Methods:** A total of 148 cases of postsurgical residual or recurrent NFPA patients were enrolled in the study. There were 111 cases with residual tumor and 37 cases with recurrent tumor. The median age was 46.0 years (Range: 10.9-75.8 years). The median tumor volume at GKRS was 3.6 cm^3^ (Range: 0.3-74.5 cm^3^), and the median tumor margin dose was 14.0 Gy (Range: 9 - 20 Gy).

**Results:** Tumor shrunk in 111 patients (75%), remained stable in 17 patients (11.5%), and progressed in 20 patients (13.5%) during a median of 64.5 months (Range: 14.5 - 236.0 months) of imaging follow-up. The progression-free survival rates were 99%, 91%, 88% and 74% at 1, 3, 5 and 10 years after GKRS, respectively. In a multivariate analysis, tumor margin dose (<13 Gy) was significantly associated with tumor progression (hazard ratio=3.526, 95% confidence interval=1.400-8.877, p=0.007). New hypopituitarism occurred in 22 out of 80 patients (27.5%), including hypogonadism (n=7), hypothyroidism (n=9), hypocortisolism (n=15) and growth hormone deficiency (n=1). In univariate and multivariate analysis, there were no factors significantly associated with new hypopituitarism. Six patients (4.1%) developed new or worsening visual dysfunction. Four patients (2.7%) developed new cranial neuropathy.

**Conclusion:** In this study, GKRS can offer a high tumor control rate as well as a low rate of complications in postsurgical residual or recurrent NFPA patients.

## Introduction

Nonfunctioning pituitary adenomas (NFPAs) do not secrete biologically active hormones and represent almost 30% of pituitary tumors 1. Usually, NFPAs may be detected because of clinical signs and symptoms such as headache, visual impairment and hypopituitarism, which are attributed to tumor compression. Transsphenoidal resection with minimal postoperative complications is widely recommended as the first-line treatment for NFPAs, especially for large tumors. Nevertheless, the incidence of recurrent tumor was reported to be 10%-20% following gross-total transsphenoidal resection for nonfunctioning macroadenomas 2-6. The tumor progression following subtotal resection without adjuvant therapy ranged from 50% to 60% 2-5. Under this circumstance, further management is generally required for residual or recurrent NFPA patients after surgical resection. GKRS has been applied in the treatment of NFPAs for decades which can provide a 10-year tumor control rate of 83%-92% as well as a low rate of hypopituitarism, varying from 9% to 32% 7-15. There are more than 26 years of experience using Gamma Knife (Elekta, Stockholm, Sweden) at the Second Affiliated Hospital of Guangzhou Medical University since 1993. To investigate the long-term outcomes and factors associated with treatment failure of GKRS for postsurgical residual or recurrent NFPA patients, we performed a single-center retrospective study.

## Methods

### Patient population

Between December 1993 and December 2016, one hundred and forty-eight cases of postsurgical residual or recurrent NFPA patients underwent GKRS at the Second Affiliated Hospital of Guangzhou Medical University were enrolled in this retrospective study. All patients had at least 12 months of follow-up. This retrospective study was approved by the institutional committee of the Second Affiliated Hospital of Guangzhou Medical University.

### Clinical and radiological evaluations

All patients were followed up with magnetic resonance (MR) imaging of the sellar, clinical and endocrinological evaluations. The follow-up evaluations were reviewed by clinicians and radiologists.

Tumor dimensions were obtained manually from MR imaging in the axial, sagittal, and coronal planes. The dimensional indices of the tumors were measured and recorded in 3 orthogonal planes: transverse (TR), anteroposterior (AP), and craniocaudal (CC). The tumor volumes were estimated using the following formula: V = (π × [TR × AP × CC])/6 16. Unfortunately, all of the digital data from model B Leksell Gamma Knife (from 1993 to 2014) in Gamma plan was missing. So, tumor volumes were calculated based on the measurement of tumor diameters. Taking into account the irregular shape of postsurgical residual or recurrent pituitary adenomas, tumor volume measurement was only a rough estimate of the actual volume. Tumor progression was defined as tumor enlargement at least 20% in tumor volume or tumor regrowth. Tumor shrinkage was defined by at least 20% shrinkage in tumor volume. Stable tumor was defined as tumor volume change within 20%. Parasellar invasion was defined as the Knosp grade 3 or 4. Suprasellar extension was defined as the tumor close to the optic nerve and chiasm (< 2 mm).

Endocrinological evaluations included measurements of cortisol, adrenocorticotropic hormone (ACTH), prolactin (PRL), growth hormone (GH), insulin-like growth factor-1 (IGF-1), thyrotropin-stimulating hormone (TSH), free triiodothyronine (FT3), free thyroxine (FT4), luteinizing hormone (LH), follicle-stimulating hormone (FSH), testosterone in men and estradiol levels in premenopausal women. New hypopituitarism was defined by requiring hormone replacement or a new deficiency in any one of the hormonal axes after GKRS. TSH deficiency was based on low FT4 (Normal Range: 12.00-22.00 pmol/L) with a low, normal or mildly elevated TSH. ACTH deficiency was defined as morning cortisol (08:00) level was <100 nmol/L. If morning cortisol level was >400nmol/L, ACTH deficiency was excluded. If morning cortisol ≥100 nmol/L and <400nmol/L, a stimulation test was used to confirm ACTH deficiency. Gonadotrophin deficiency in males was defined by low serum testosterone levels (Normal Range: 0.18-0.78 nmol/L) without elevated LH and FSH levels. Gonadotrophin deficiency in females was defined by amenorrhea with low serum estradiol and low gonadotrophins in premenopausal females, and the absence of high gonadotrophins (LH and FSH) in menopausal women. GH deficiency was based on a low age- and sex-adjusted IGF-1 or an insufficient rise in GH levels during a stimulation test. As many patients were from long distances and had poor compliance, some patients did not routinely follow up with endocrine evaluation and dynamic testing.

### Gamma knife radiosurgery technique

Model B Leksell Gamma Knife Unit was performed from 1993 to 2014 and Perfexion Unit (Elekta Instrument, Inc.) thereafter. Thin-slice stereotactic MR imaging with contrast was performed with axial and coronal images through the sella following stereotactic Leksell frame placement. Subsequently, the planning of GKRS treatment was made in consultation with a medical physicist, radiation oncologist and neurosurgeon. The prescribed dose was adjusted depending on tumor volume, distance to the optic nerve and chiasm, and history of previous radiotherapy. The maximal dose to the optic nerve and chiasm was restricted to 9 Gy, and to the lateral wall of the cavernous sinus was restricted to 15 Gy. Small collimators of 4 mm and 8 mm were mainly adopted to achieve better GKRS treatment plan conformality.

### Statistical analysis

The prognostic value of different variables relative to tumor control and new hypopituitarism were confirmed by univariate and multivariate analysis. Log-rank test statistics and a step forward likelihood ratio method of Cox proportional hazard models were applied for univariate analysis and multivariate analysis respectively. Kaplan-Meier curves were plotted for progression-free survival (PFS) and new hypopituitarism. Probability values <0.05 were defined as statistically significant. For statistical analysis, IBM's SPSS (version 21.0) was used.

## Results

### Patient characteristics

One hundred and forty-eight patients who had undergone GKRS for postsurgical residual or recurrent NFPAs between December 1993 and December 2016 were selected from the single-center. The patient population consisted of 80 male (54.1%) and 68 female (45.9%) patients with a median age of 46.0 years (Range: 10.9-75.8 years). The median follow-up was 64.5 months (Range: 14.5-236.0 months). There were 111 patients (75%) with residual tumor after surgery and 37 patients (25) with recurrent tumor. The median time between first surgery and GKRS was 6.0 months (Range: 0.9 - 326.7 months). The median tumor volume at GKRS was 3.6 cm^3^ (Range: 0.3 - 74.5 cm^3^). There were 140 patients (94.6%) with suprasellar extension and 77 patients (52.0%) with parasellar invasion before surgical resection. Only one patient had previous radiotherapy. Ninety-four patients (63.5%) had visual dysfunction. There are 15 patients (10.1%) who presented with cranial nerve (CN) dysfunction before GKRS (Table 1).

The median tumor margin dose was 14 Gy (range, 9-20 Gy) at a median prescription isodose 40% (Range: 30-71%). The median maximum dose was 33 Gy (Range: 14-50 Gy) (Table 1).

### Tumor control

**Figure 1:**
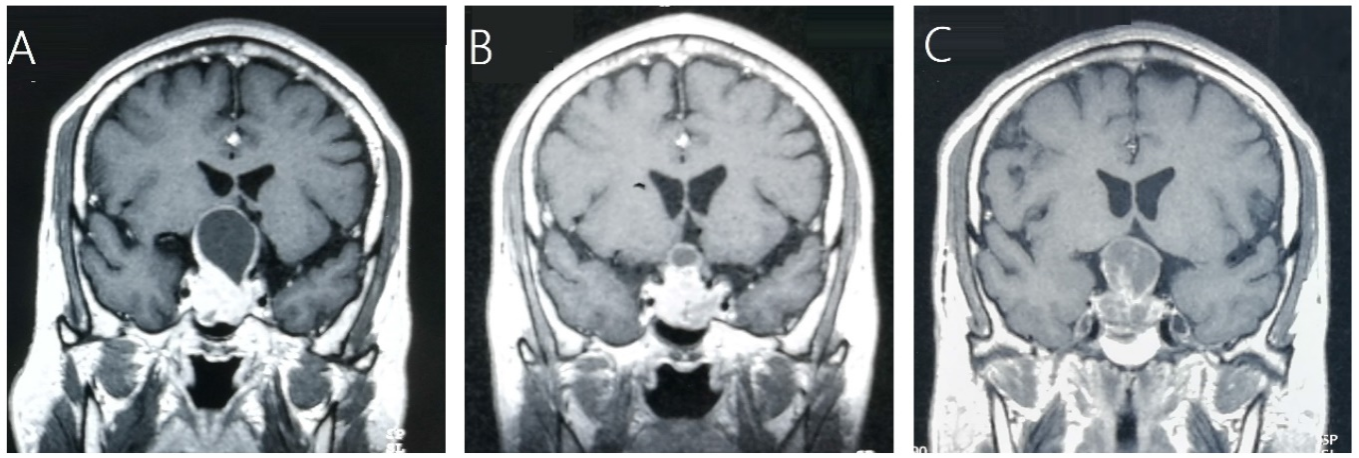
A 45-year-old male patient with residual NFPA received GKRS (10.5Gy/30%) at 4.9 months after surgery and tumor progression due to progressive cystic enlargement was detected at 32.5 months after GKRS. **A,** MRI showed pituitary giant adenoma in sellar area. **B,** MRI showed residual tumor at 4.9 months after surgery. **C,** MRI showed tumor enlargement due to progressive cystic enlargement at 32.5 months after GKRS.

**Figure 2:**
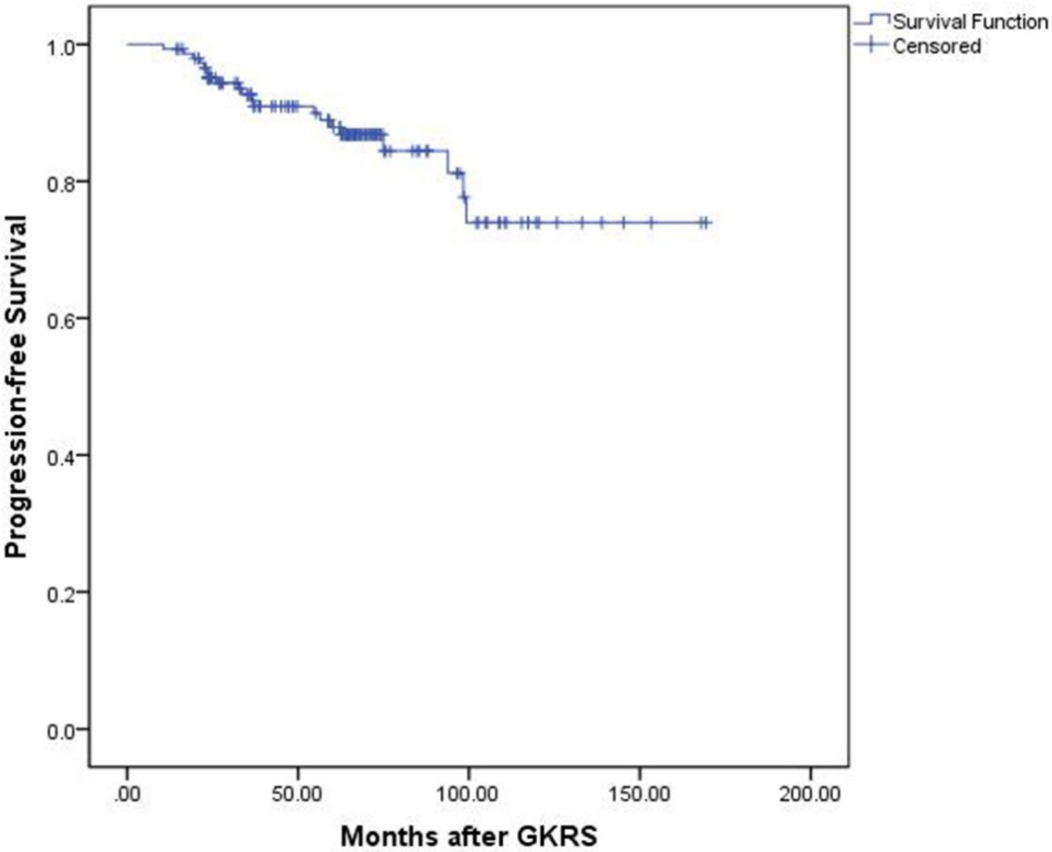
Kaplan-Meier curve of progression-free survival for the entire series.

Tumor control was confirmed by follow-up MR imaging in 128 patients (86.5%). The data was shown in Table 2. Tumor shrank in 111 patients (75%) and remained stable in 17 patients (11.5%). Tumor progression was confirmed in 20 patients (13.5%) (Figure 1). The median time of tumor progression was 35.1 months (Range: 10.7 - 99.2 months) after GKRS. Of the 16 patients with progression due to tumor regrowth, 9 patients underwent repeat GKRS, 2 patients underwent surgery, 2 patients underwent close observation, and 3 patients were lost to follow-up. Of the 4 patients with progressive cystic enlargement, 2 patients underwent surgery, 2 patients underwent close observation. The progression-free survival rates were 99%, 91%, 88% and 74% at 1, 3, 5 and 10 years after postsurgical GKRS respectively (Figure 2).

**Figure 3:**
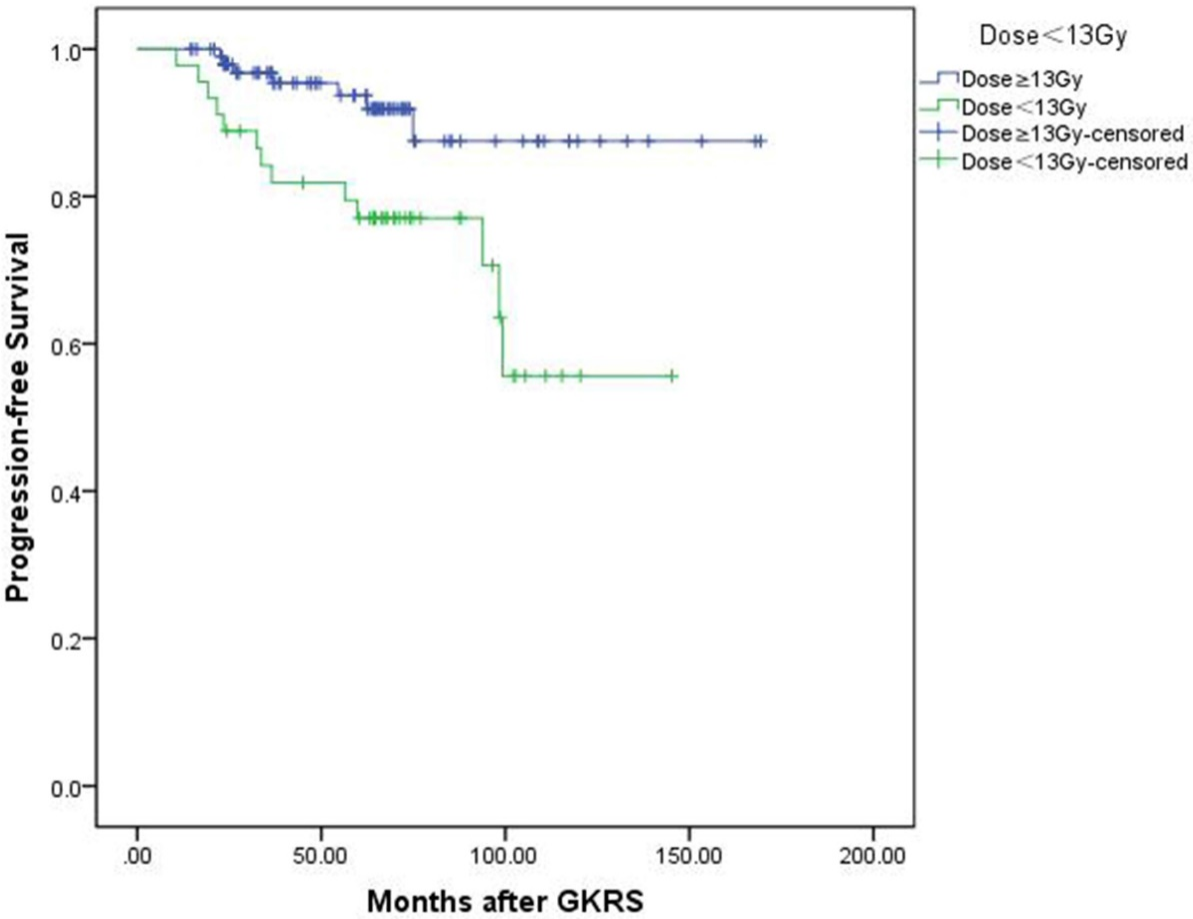
Kaplan-Meier curve of progression-free survival of tumor margin dose <13 Gy VS ≥13 Gy. Low tumor margin dose (<13 Gy) shows shorter progression-free survival. (p=0.004).

In univariate analysis, factors associated with shorter PFS included parasellar invasion (p=0.034) and tumor margin dose (<13 Gy) (p=0.004). In multivariate analysis, only tumor margin dose was significantly associated with tumor progression (hazard ratio [HR] = 3.526, 95% confidence interval [CI] = 1.400-8.877, p=0.007) (Table 3). Kaplan-Meier plot generated for PFS (tumor margin dose <13 Gy VS tumor margin dose ≥13Gy) was shown in Figure 3.

### Hormonal outcomes

**Figure 4:**
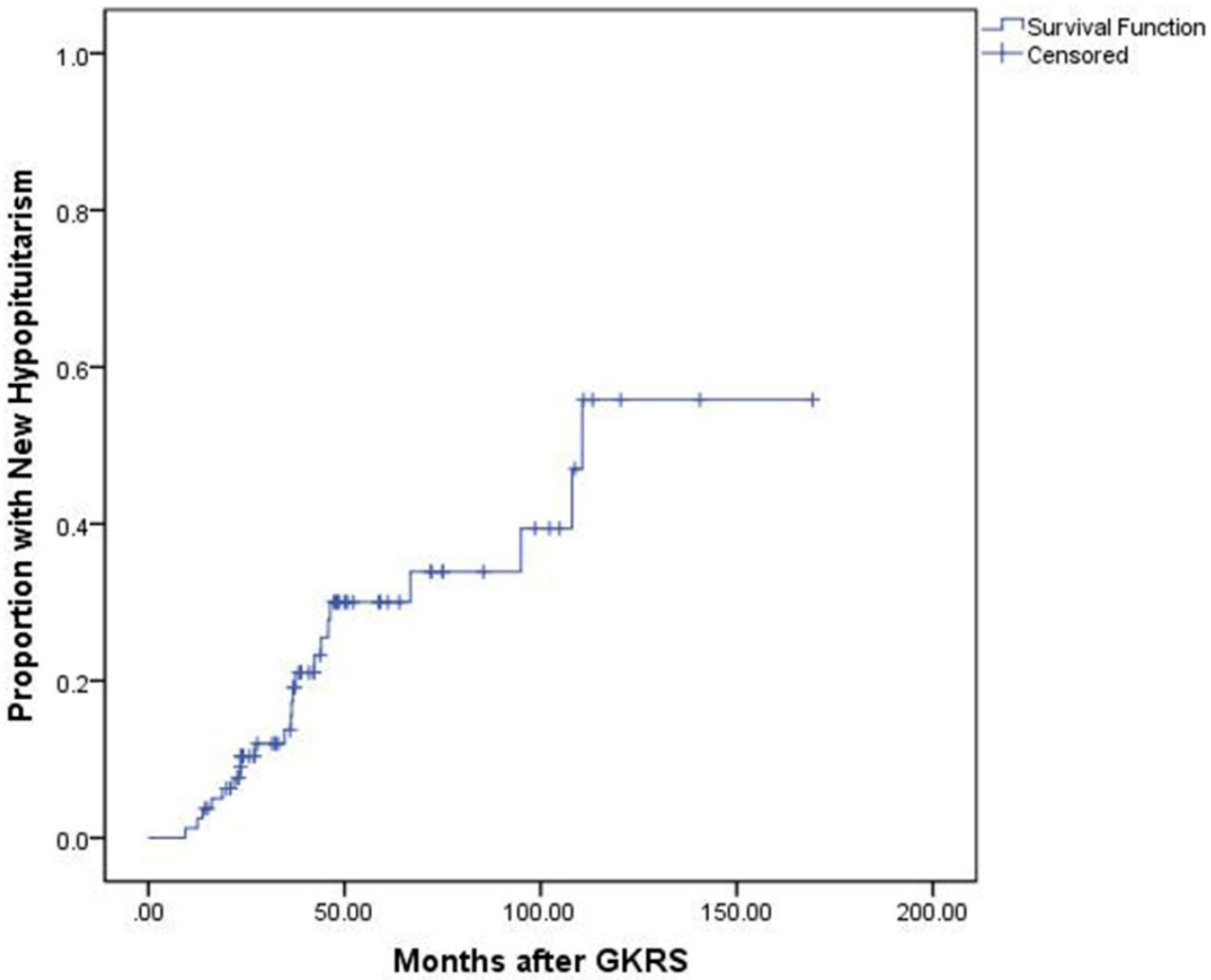
Kaplan-Meier curve of proportion with new hypopituitarism for the entire series.

The median endocrine follow-up was 60.6 months (Range: 14.5-169.4.0 months). Of the 80 patients with complete endocrine data before and after GKRS, 51 patients (63.8%) presented with hypopituitarism before GKRS, including hypogonadism (n=48), hypothyroidism (n=15) and hypocortisolism (n=11). Of the 29 patients (36.3%) with normal endocrine function before GKRS, 7 patients (24.1%) developed completely new hypopituitarism, including hypogonadism (n=5), hypothyroidism (n=4) and hypocortisolism (n=3). The median time for completely new hypopituitarism in these patients was 37.56 months (Range: 16.2-66.8 months). Finally, of the 80 patients, 22 patients (27.5%) developed new hypopituitarism, including hypogonadism (n=7), hypothyroidism (n=9), hypocortisolism (n=15) and GH deficiency (n=1) (Table 2). The median time until detection of new hypopituitarism after GKRS was 36.5 months (Range: 9.4 - 110.6 months). The cumulative rates of developing new hypopituitarism at 1, 3, 5 and 10 years was 4%, 21%, 30% and 57%, respectively (Figure 4).

In univariate and multivariate analysis, there were no factors significantly associated with new hypopituitarism (Table 3).

### Clinical outcomes

After GKRS, clinical outcomes, including visual and cranial nerve function, were evaluated. Six patients (4.1%) worsened in visual function due to compression of chiasm by tumor progression after GKRS. However, visual dysfunction may also be influenced by nature of the visual dysfunction and GKRS. Finally, 4 patients (2.7%) suffered new cranial dysfunction after GKRS (two patients with CN V dysfunction, one patient with CN VI dysfunction, another patient CN III, IV, and VI dysfunction). All 4 patients had large tumors with parasellar invasion. Tumor shrinkage was observed in three patients and of these patients with CN dysfunction, these may be caused by GKRS. Tumor progression and invasion into the cavernous sinus and retrobulbar space subsequently caused CN III, IV, and VI dysfunction in another patient (Table 2).

## Discussion

NFPAs are often detected due to symptoms and clinical signs. Transsphenoidal surgical resection with minimal complications is the recommended first-line treatment for most symptomatic patients. It has the advantages of rapid decompression of surrounding structures and pathological analysis. However, for tumors with suprasellar extension and parasellar invasion, complete surgical resection may be difficult to achieve (46%-75% of all surgical patients) 6, 17, 18. The incidence of recurrent tumor after gross total resection occurred in 10%-20% of cases 2-6. For patients following subtotal resection without adjuvant therapy, the rate of tumor progression ranged from 50% to 60% 2-5. In a series of 126 postsurgical residual NFPA patients, Gittoe et al 19 reported progression free survival rates of 93% at 5, 10 and 15 years for patients with residual NFPAs treated with RT after surgery, and 68%, 47% and 33% for those patients with observation, respectively. Other studies 2, 20 also reported similar results. Given the high rate of recurrent or residual NFPAs after surgical resection and tumor progression after surgery without radiotherapy, further management is generally required.

### Radiation techniques for postsurgical residual or recurrent NFPAs

Radiation techniques have developed from 3D conformal radiotherapy (3D-CRT) to stereotactic radiosurgery (SRS) or fractionated stereotactic radiotherapy (FSRT). In previous studies of postsurgical conventional radiotherapy for NFPAs, tumor control rates were 80-90% at 10 years and 75-90% at 20 years with doses ranging from 45 to 55 Gy at 1.8-2.0 Gy per fraction 21. The hypopituitarism occurred in 20% to 30% at 5 years after conventional radiotherapy^21^. SRS or FSRT which has advantages of precise tumor localization and a better dose conformity than conventional radiotherapy is the most commonly used radiation techniques and essential part in the treatment of pituitary adenomas. As GKRS is still considered the gold standard of SRS, GKRS is the most reported in literatures. For FSRT, tumor control rate was around 95% at 5 years and new hypopituitarism was reported in 10% to 48% in NFPA patients with doses ranging from 45-54 Gy at 1.8-2.0 Gy per fraction 21. GKRS has been proved to offer a high tumor control rate of 83%-92% and a low rate of 9%-32% of new hypopituitarism for pituitary adenomas 8, 9, 11, 14, 22, 23. Tumor local control and complications were similar between GKRS and FSRT. However, the superiority of GKRS over FSRT for NFPA patients according to tumor control and complications has yet to be defined.

### Timing of radiosurgery

The effect of timing of radiosurgery for residual or recurrent NFPAs had been discussed in previous reports. Pomeraniec et al. 24 conducted a multicenter retrospective study to evaluate the effect of timing of radiosurgery on outcomes. In this study, early GKRS (≤6 months after resection) was related with a lower risk of radiological progression compared with delayed radiosurgery (>6 months after resection). What's more, the rate of delayed endocrinopathy was not related to the timing of radiosurgery. In the study of Sadik et al. 25, adjuvant radiosurgery (≤6 months after surgery) also provided similar tumor control rate with delayed GKRS, but there was a trend that adjuvant GKRS may provide a fewer endocrinologic deficits. It seemed that early GKRS should be used for residual NFPAs after surgery. In the current study, the timing of GKRS (≤6 months after surgery versus >6 months after surgery) was not associated with tumor progression (p=0.516) and new hypopituitarism (p=0.336). Tumor characteristic (residual VS recurrent) was not related with tumor progression (p=0.695) and new hypopituitarism (p=0.963). As a delayed GKRS for postsurgical residual NFPAs would increase the risk of tumor regrowth and visual dysfunction, we suggested early GKRS should be used after surgical resection.

### Tumor control and related risk factors with GKRS

Adjuvant GKRS has been proved to offer a high tumor control rate of 83%-92% for pituitary adenomas, as well as a low risk of complications. Gopalan et al. 14 reported a tumor control rate of 83% in 45 NFPA patients after GKRS. Lee et al. 22 reported 41 NFPA patients underwent GKRS as primary management. The overall tumor control rate was 92.7%, and the tumor control rate was 94% and 85% at 5 and 10 years postradiosurgery, respectively. In the current study, 148 cases of postsurgical residual or recurrent NFPA patients underwent GKRS. The median tumor margin dose was 14 Gy (Range: 9-20 Gy). The progression-free survival rates were 99%, 91%, 88% and 74% at 1, 3, 5 and 10 years after postsurgical GKRS, respectively, which was similar with other studies.

There are many potential risk factors associated with tumor control, such as tumor margin dose, tumor volume, parasellar invasion and suprasellar extension. Gopalan et al. 14 reported that tumor volumes greater than 5 ml at the time of GKRS were significantly associated with tumor growth (p=0.003) based on 48 NFPA patients treated with GKRS. They also noted tumor margin doses less than 12 Gy was significantly associated with a lower tumor control rate (33%) relative to doses of 12 Gy or higher (80%). In the study of Park et al. 26, lower marginal dose (<14 Gy), larger tumor volume (≥4.5 ml), and ≥2 prior recurrences were associated with shorter PFS in univariate analysis based on 125 NFPA patients undergoing GKRS; larger (≥4.5 ml) tumor volume (p =0.04; HR = 5.413), and ≥2 prior recurrences (p =0.01; HR = 5.777) were related with shorter PFS in multivariate analysis. Sun et al. 8 reported 204 cases of residual or recurrent NFPA patients treated with GKRS. In the study, parasellar invasion affected tumor control significantly after adjusting for all other covariates (HR=3.705, 95% CI=1.248-11.000, p=0.018). In our study, parasellar invasion and tumor margin dose were related with tumor control in univariate analysis. Only tumor margin dose was independently related with tumor control (HR=3.526, 95% CI=1.400-8.877, p=0.007) in multivariate analysis. There were 140 patients (94.6%) with suprasellar extension, and 77 patients (52%) with parasellar invasion in this study. The average tumor volume was 6.5 ml. Large tumor volume, parasellar and suprasellar extension were risk factors for tumor progression. A relative low tumor margin dose (<13 Gy) was not enough for tumor control in those patients with so many risk factors. For residual or recurrent NFPA patients with large tumor volume, repeat surgery should be considered to reduce tumor volume as much as possible.

### New hypopituitarism after GKRS and related risk factors

Hypopituitarism is the most common complication after GKRS for pituitary adenoma patients. Previous reports showed the rate of new hypopituitarism at almost 9%-32% 8-13, 23. Risk factors such as tumor volume, tumor margin dose, higher pituitary gland dose and history of radiation, were potentially related with new hypopituitarism. Pollock et al. 13 reported the rate of new hypopituitarism was 18% and 50% at 5 years after SRS for tumors volume ≤4 cm^3^ and >4 cm^3^ respectively. In the study of Lee et al. 22, tumor margin dose >18 Gy and a maximum dose >36 Gy were independent prognostic factors related with hypopituitarism. Graffeo et al. 27 found a lower rate of hypopituitarism in patients with a mean gland dose <11 Gy. The rate of hypopituitarism at 2-years and 5-years were 2% and 5% for a mean gland dose <11 Gy, whereas the rate of hypopituitarism was 31% and 51% at 2-years and 5-years for patients with a mean gland dose ≥11 Gy. A correlation was also found between prior radiotherapy and new hypopituitarism after SRS in the study of Park et al. 26. However, in our study, no factors were significantly correlated with new hypopituitarism and the rate of new hypopituitarism was similar to other studies. From what was discussed above, compression by tumor mass and radiation dose exposition to the normal pituitary gland were the main causes of new hypopituitarism after GKRS in patients with NFPA. Hence, decompression of pituitary gland by primary total or subtotal surgical resection and dose reduction in pituitary gland without sacrificing radiation dose to tumor may be contributed to reduce the rate of new hypopituitarism after GKRS for patients with pituitary adenomas.

### Other complications with GKRS

The incidence of neurological complications such as visual impairment and cranial nerve dysfunction were relatively low after GKRS, which was reported to be between 0% and 5% 11, 23, 26. In this study, six patients (4.1%) developed new or worsening visual dysfunction. Four patients (2.7%) developed new or worsening cranial neuropathy. Visual dysfunction and cranial nerve dysfunction within the cavernous sinus could be caused by compression and radiation damage, especially in patients with suprasellar invasion and parasellar extension. The optic pathway was generally restricted to <8 Gy in GKRS 28. That may sacrifice radiation dose to the tumor near the optic pathway. The dose of up to 40 Gy appears to be a relatively safe to the cranial nerves of the cavernous sinus 28.

### Study limitations

In this study, some limitations should be noted. First, this was a retrospective study and therefore contained information and selection biases. Second, taking into account the irregular shape of postsurgical residual or recurrent pituitary adenomas, tumor volume measurement was only a rough estimate of the actual volume. Third, as some patients were from long distances and had poor compliance, many patients did not routinely follow up with endocrine evaluations; only 80 patients (54.1%) with complete endocrine evaluations before and after GKRS were analyzed for new hypopituitarism, the small number of cases may limit statistical power. Four, although all patients were histologically confirmed as NFPAs, there was a lack of pathological markers of aggressive behavior, which may be associated with tumor regrowth. Finally, given the lack of IGF-1 or a stimulation test in some patients followed up in rural area, GH deficiency may be under estimated.

## Conclusion

Surgical resection is the recommended first-line treatment for NFPA patients. For residual and recurrent NFPA patients after surgery, GKRS has been used for decades. In this study, GKRS can offer a high tumor control rate as well as a low rate of complications in postsurgical residual or recurrent NFPA patients.

## Figures and Tables

**Table 1:** Characteristics of 148 patients with postsurgical residual or recurrent nonfunctioning pituitary adenomas and GKRS parameters

Characteristic	Value
Male/Female, n (%)	80/68 (54.1/45.9)
Median age, (range), years	46.0 (10.9-75.8)
Median FU length, (range), months	64.5 (14.5-236.0)
Prior surgery, once, n (%)	125 (84.5)
Twice, n (%)	22 (14.9)
Three times, n (%)	1 (0.7)
**Tumor type, n (%)**	
Residual tumor	111 (75)
Recurrent tumor	37 (25)
Months between 1st surgery and GKRS, median (range)	6.01 (0.9-326.7)
Median tumor volume at GKRS, (range), cm^3^	3.6 (0.3-74.5)
Parasellar invasion, n (%)	77 (52.0)
Suprasellar extension, n (%)	140 (94.6)
Previous radiotherapy, n (%)	1 (0.7)
**Visual function before GKRS, n (%)**	
Visual field defect and/or visual acuity decrease	94 (63.5)
Normal	54 (36.5)
Pre-GKRS cranial nerve dysfunction, n (%)	15 (10.1)
**GKRS parameters**	
Median tumor margin radiation dose, (range), Gy	14 (9-20)
Median maximum radiation dose, (range), Gy	33 (14-50)
Median prescription isodose, (range), %	40 (30-71)

Abbreviations: FU, follow up; GKRS, gamma knife radiosurgery.

**Table 2:** Imaging and clinical outcomes of 148 patients with postsurgical residual or recurrent nonfunctioning pituitary adenomas

Outcomes	Value, n (%)
Tumor control	128 (86.5)
Shrinkage	111 (75)
Stability	17 (11.5)
Progression	20 (13.5)
Visual function worsened	6 (4.1)
New CN dysfunction	4 (2.7)
New hypopituitarism after GKRS	22 (27.5)*
Hypogonadism	7
Hypothyroidism	9
Hypocortisolism	15
GH deficiency	1

Abbreviations: CN, cranial nerve. *Eighty patients with sufficient endocrine evaluation data were available for analysis of new hypopituitarism.

**Table 3:** Results of univariate and multivariate analysis for tumor control and new hypopituitarism after GKRS

Variables	Tumor control				New hypopituitarism
	Univariate, *p*	Multivariate, *p*	HR	95% CI	Univariate, p
Age (≥55 years)	0.534	NA	NA	NA	0.721
Sex (male VS female)	0.299	NA	NA	NA	0.849
Parasellar invasion	0.034^*^	0.082	NA	NA	0.903
Suprasellar extension	0.806	NA	NA	NA	0.695
Tumor margin dose (<13 Gy)	0.004^*^	0.007^*^	3.526	1.400-8.877	0.529
Tumor volume (≥4 cm^3^)	0.255	0.932	NA	NA	0.545
Tumor type (recurrent VS residual)	0.695	NA	NA	NA	0.963
Tumor response	NA	NA	NA	NA	0.069

Abbreviations: GKRS, gamma knife radiosurgery; CI, confidential interval; HR, hazards ratio; NA, not available. *Statistically significant (P <0.05).
